# Defining rapid early progression in glioblastoma—A volumetric approach

**DOI:** 10.1093/noajnl/vdag153

**Published:** 2026-06-08

**Authors:** Vikram Munlapudi, Jacob Guzzino, David J Crompton, Shubhi Agarwal, Eric J Lehrer, Shelby Kern, Aaron Bogan, Sujay A Vora, Alfredo Quinones-Hinojosa, Terry C Burns, Wendy J Sherman, Paul D Brown, Nadia Laack, Jennifer Peterson, Joon H Uhm, Michael W Ruff, Ugur T Sener, William G Breen, Daniel M Trifiletti

**Affiliations:** Department of Radiation Oncology, Mayo Clinic Florida, Jacksonville, Florida, USA; Department of Radiation Oncology, Mayo Clinic Florida, Jacksonville, Florida, USA; Department of Radiation Oncology, Mayo Clinic Florida, Jacksonville, Florida, USA; Department of Radiation Oncology, Mayo Clinic Florida, Jacksonville, Florida, USA; Department of Radiation Oncology, Mayo Clinic Rochester, Rochester, Minnesota, USA; Department of Radiation Oncology, Mayo Clinic Rochester, Rochester, Minnesota, USA; Department of Research Biostatistics, Mayo Clinic Arizona, Phoenix, Arizona, USA; Department of Radiation Oncology, Mayo Clinic Arizona, Phoenix, Arizona, USA; Department of Neurosurgery, Mayo Clinic Florida, Jacksonville, Florida, USA; Department of Neurosurgery, Mayo Clinic Rochester, Rochester, Minnesota, USA; Department of Neurology and Oncology, Mayo Clinic Florida, Jacksonville, Florida, USA; Department of Radiation Oncology, Mayo Clinic Rochester, Rochester, Minnesota, USA; Department of Radiation Oncology, Mayo Clinic Rochester, Rochester, Minnesota, USA; Department of Radiation Oncology, Mayo Clinic Florida, Jacksonville, Florida, USA; Department of Neurology and Oncology, Mayo Clinic Rochester, Rochester, Minnesota, USA; Department of Neurology and Oncology, Mayo Clinic Rochester, Rochester, Minnesota, USA; Department of Neurology and Oncology, Mayo Clinic Rochester, Rochester, Minnesota, USA; Department of Radiation Oncology, Mayo Clinic Rochester, Rochester, Minnesota, USA; Department of Radiation Oncology, Mayo Clinic Florida, Jacksonville, Florida, USA

**Keywords:** glioblastoma, rapid early progression, residual tumor, volumetric analysis

## Abstract

**Background:**

The current treatment of glioblastoma involves resection followed by radiotherapy (RT) and temozolomide. Rapid early progression (REP) of tumor in the surgery-to-radiotherapy interval (SRI) has been described using categorical or semi-quantitative assessments and is associated with inferior clinical outcomes. This study seeks to establish and validate a quantitative definition for REP and examine how REP could inform future therapy.

**Methods:**

Volumes of tumor and resection cavity at 3 time points—preoperative, postoperative and RT-planning (pre-RT)—were contoured among patients who had undergone standard of care. A minimal, but clinically appreciable, threshold for defining REP based on change in tumor volume was established, validated, and subsequently used to assess actionable predictors of REP and its prognostic value.

**Results:**

An increase of >2 cc in volume or a new satellite tumor between the post-operative to planning MRI was found to be a sensitive, pragmatic, and statistically significant cutoff for REP (*P* = .002). Actionable predictors such as SRI of 6 weeks and *a* > 5 cc residual immediate post-operative tumor volume were found to be statistically significant predictors of REP. REP was also found to be a statistically significant prognostic indicator for overall survival (*P* = .002) and progression-free survival.

**Conclusion:**

These findings support that an increase in tumor volume between post-operative and pre-RT MRI of >2 cc is the ideal working definition of REP in this clinical setting. Furthermore, REP could serve as a prognostic ­biomarker to track treatment response. Additionally, 2 actionable predictors of REP, post-op residual tumor volume and SRI, are identified.

Key Points

- A >2 cc change in tumor volume was the most optimal cutoff for rapid early progression (REP).
- REP could be a marker for treatment response in glioblastoma.
- Controlling post-operative residual volume and surgery-to-radiotherapy interval might minimize REP.

Importance of the Study

Although prior studies have examined how rapid early progression (REP) can lead to poorer clinical outcomes like overall survival and progression-free survival, few have defined REP in this setting between surgery and radiation therapy. Current criteria for progression of disease by Response Assessment in Neuro-Oncology are validated for the post-treatment setting, hence, this study sought to quantitatively define REP in the mid-treatment setting using a large multi-center patient population. Furthermore, predictors of REP, surgery-to-radiotherapy interval, and postoperative residual volumes were identified that could be acted upon in the clinical setting and improve outcomes in this subset of the patient population. Future studies could use this definition not only as a marker for treatment response but also as a method of identifying additional actionable risk factors.

Glioblastoma accounts for 52.2% of malignant CNS tumors and 13.7% of all CNS tumors, second only to meningiomas.^1^ It is considered very aggressive and associated with a poor prognosis with a median survival rate of 15 months.^2^ Glioblastoma is a WHO grade IV glioma determined both histologically and, since 2021, with molecular features (isocitrate dehydrogenase (IDH), telomerase reverse transcriptase (TERT), and epidermal growth factor receptor (EGFR)) even without appropriate histological features.^3^

The current standard of care is 3-fold and aims for maximal safe resection of the tumor followed by radiotherapy (RT) and chemotherapy with concurrent TMZ beginning approximately 4-6 weeks after surgery based on current practice guidelines.^4-15^ Despite the limited advances over recent decades, with the most recent being tumor-treating fields in 2015, treatment is far from optimized, and post-radiotherapy tumor recurrence typically occurs within 12 months.^2^ Genetic factors such as IDH mutations and O^6^-methylguanine-DNA methyltransferase (MGMT) methylation status have demonstrated a significant role in the efficacy of RT and chemotherapy and overall prognosis.^5^^,^^6^

Although gross resection of enhancing disease can sometimes be achieved radiologically, microscopic remnants and tendrils can exist beyond the resection borders.^7^ This is the principle behind administering RT and chemotherapy post-operatively, targeting residual microscopic tumor cells. During the surgery-to-radiotherapy interval (SRI) there can be new growth in as many as 52% of cases, associated with inferior prognosis. This phenomenon is termed rapid early progression (REP).^8^

REP was first studied by Pirzkall et al in which they examined imaging in 32 patients at various time points, of which 17 patients had new contrast enhancement consistent with new tumor growth. These patients had a median survival rate of 14.6 compared to the 24 months in those without.^10^ REP has been proposed as a potential phenotypic prognostic factor when treating glioblastoma and could augment clinical decision making by serving as an early measure of disease response.

Since then, multiple studies have examined the relation between REP and extent of resection (EOR) with poor overall survival (OS) and progression-free survival (PFS).^8^^,^^10^^,^^11^,^13^ However, these have principally relied on relatively small data sets and defined REP based on semi-quantitative or categorical measurements.^11^^,^^14^ This definition of REP has limited reproducibility and external validity. One such study examined both predictors of REP, SRI, and residual enhancing tumor, as well as REP’s prognostic value. Although they found REP to be associated with lower median survival as well as an increase in risk of interim progressive disease by 3.9% for every 1 cc increase in residual and 8.1% for every 1 day prolongation of SRI, their definition of REP relied on Response Assessment in Neuro-Oncology (RANO) criteria, which has been validated in the post-RT setting but not in the pre-RT setting.^11^ To date, while REP is a known prognostic factor, there are no validated methods reported to define REP in this setting. The aim of this study is to establish and validate a quantitative definition of REP based on volumetric analysis.

## Methods

This retrospective, IRB-approved study screened a cohort of 587 patients with histopathologically confirmed WHO grade IV glioma (2016) who have undergone maximal safe resection of any extent (gross total resection [GTR], near-total resection [NTR], subtotal resection [STR]) followed by RT and concurrent temozolomide at a multisite academic center between the years 2014 and 2023. All patients in this study were treated in a single hospital system across major clinical sites: Site A (*n* = 190), Site B (*n* = 69), and Site C (*n* = 29). An additional 30 patients were included from affiliated community-based hospitals and practices included in the health system. The characteristics of this database such as age, tumor location, MGMT methylation status, sex, time to pre-RT imaging, and Eastern Cooperative Oncology Group (ECOG) scores were obtained via review of patient electronic medical records. PFS following RT was defined using the RANO criteria for progression: (i) >25% increase in summation of products of perpendicular diameters, (ii) significant increase in non-enhancing Flair/T2W lesions, and (iii) new lesions. PFS and OS were measured from the date of initial surgery. Bidimensional products (BPD) were used to assess specifically for PFS following treatment, whereas volumetric criteria were assessed to define REP in the SRI.

Further inclusion criteria included patients older than 18 years, receipt of RT within the multisite health system, and availability of T1-post contrast images at pre-op, post-op, and pre-RT intervals. Typically, patients underwent postoperative MRI within 24 h of resection and RT planning MRIs < 2 weeks prior to RT. Patients were otherwise excluded if they had previously undergone RT, were missing key image timepoints, or if the images were obtained as CT imaging studies only.

**Figure 1.**
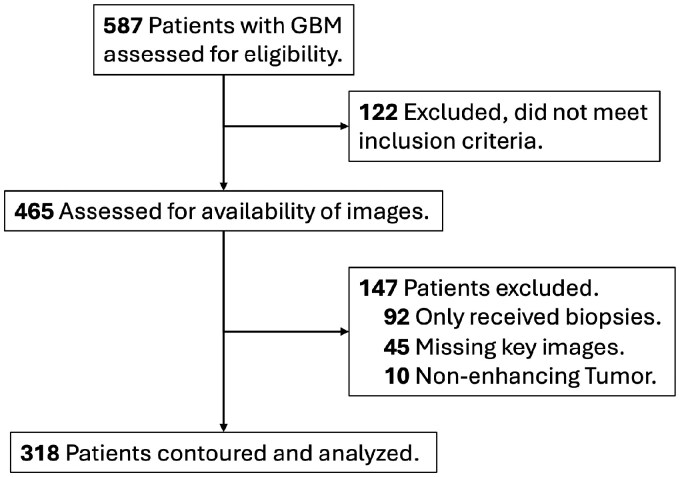
Flow diagram of patient selection in the study cohort. Patients were screened and excluded based on pre-defined criteria.

None of the patients in the database that had undergone biopsy alone had a post-op MRI available and hence were all excluded. The final analytic sample consisted of 318 patients (Figure 1).

### Imaging Analysis

**Figure 2.**
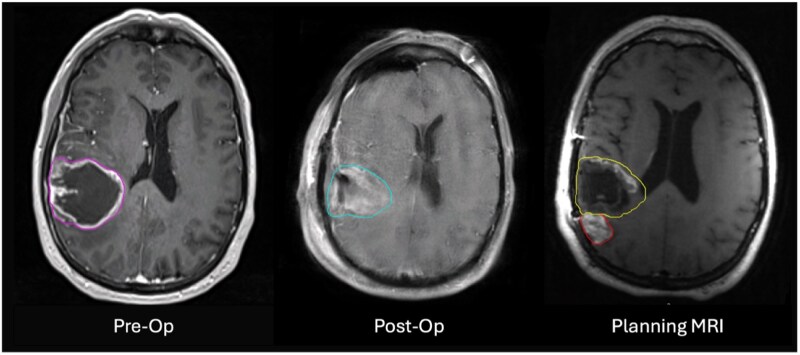
T1 gadolinium enhanced MRIs were used to contour tumor and cavity volumes at 3 time points: pre-operative, post-operative and radiotherapy (RT) planning. Pictured above are the Pre-operative GTV (left), post-operative cavity (center), planning cavity and GTV/rapid early progression (REP) (right). On the planning MRI, the cavity contour is shown superiorly while REP is shown inferiorly. REP here is defined as an increase in enhancing tumor volume between post-operative and pre-RT imaging (≥2 cc). The region of REP represents this increase enhancing tumor. Slices were aligned using anatomical landmarks, slight variations in positions may be due to post-operative changes such as mass effect.

Using volumetric T1 gadolinium-enhanced images, volumes of tumor and resection cavity at 3 time points—preoperative, postoperative, and pre-RT—were contoured for each patient using MIM software (Figure 2). Segmentations were ­performed by a single human reviewer blinded to patient outcomes using a slice-by-slice approach, with tumor boundaries defined by areas of nodular contrast enhancement. Linear or diffuse enhancement across the resection cavity, postoperative changes and blood products were all excluded. More indeterminate tumor volumes reviewed by a second reviewer. Indeterminate cases were defined as those with uncertainty in delineating tumors from postoperative or treatment-related changes, particularly in cases involving postoperative ischemia or complex anatomical changes following significant debulking. These were reviewed with the principal investigator. Final segmentations were determined by consensus, with earlier cases revisited as needed to ensure consistent application of segmentation criteria.

For the pre-operative MRI, either stereotactic pre-op surgery planning MRIs were used when available or any MRI as close as possible to the date of operation. Then, an initial tumor volume was contoured (preop-gtv, gross tumor volume). A postoperative MRI, typically within 48 h of resection, was used to contour the cavity (postop-cavity) as well as any residual enhancing tumor in the case of near-total or subtotal resection (postop-gtv). EOR was defined as either GTR (no measurable residual enhancing tumor), NTR; ≥ STR (<90%). EOR was based on radiographic estimation rather than volumetric segmentation. This was determined using early postoperative contrast-enhanced MRIs (within 6 days of surgery) and the degree of residual nodular enhancement was used to classify the resection.

For the pre-RT MRI scans, MRIs at the time of RT simulation were used to draw volumes for the resection cavity as well as any enhancing tumor. In some cases, there was another MRI taken closer to the beginning of RT, typically due to an adverse health event in the interim, in which case this MRI was used for volumetrics as it was used for RT planning.

Although there were subtle differences in MRI techniques between the various clinical sites and imaging locations based on site-specific protocols, all MRI’s used for segmentation were T1-weighted post-contrast sequences obtained isotropically, using field strengths between 1.5 and 3T. Most postoperative imaging MRIs were acquired on standard clinical MRI systems, although a subset of patients underwent intraoperative MRI. Slice thickness also varied between patients and time points but, in general, were 1.20 mm thick. To maintain comparability, segmentations were performed using standardized criteria applied consistently across all time points.

### Statistical Analysis

Analysis of patient demographics, outcomes (OS and PFS), and REP was performed using IBM SPSS Statistics. Patient characteristics including sex, age, MGMT status, ECOG scores, time to pre-rt imaging (defined as surgery date to RT simulation interval [SRI]), EOR, and location of tumor were analyzed for association with REP using chi-squared test with binary logistic regression to calculate odds ratio (OR). Univariate screening was used to identify predictors of REP, OS, and PFS.

As there currently are no established guidelines for determining progression of disease in the interval between surgery and RT, various cutoffs of new enhancement were analyzed via univariate and multivariate analysis to determine the optimum threshold. Both percent change in tumor volume from postoperative MRI to planning MRI as well as gross change in cubic centimeters (cc) were evaluated. Median values of gross change (2.44 cc) were tested as well as various other thresholds (1 cc-10 cc and 5%-20% in increments of 5%). For percent change and cc change, REP was defined as an increase in X% or X ccs, respectively, from post-operative gross tumor volume (GTV) to pre-RT GTV. However, there were 2 exceptions in which enhancement of any volume was classified as REP regardless of cutoff criteria: (i) if the patient had undergone a GTR with no residual tumor on post-surgical imaging and a new tumor developed of sufficient volume for diagnostic confidence and (ii) appearance of new satellite lesions following surgery. This included new enlargements of any cc of volume. Additionally, local REP was defined as progression contiguous with the original tumor site or resection cavity and included both enlargement of residual tumor and new enhancement at the margins. Distant or satellite REP was defined as any new enhancing lesions without contact with these regions.

The samples were then classified with either the absence or presence of REP based on either a given cutoff or the above exceptions. These were then evaluated for prediction of OS via Kaplan-Meier survival curve using a log-rank (Mantel-Cox) test for univariate analysis.

A screen of chi-square tests was then used to identify the most significant, actionable, residual volume (on post-op) associated with REP by dichotomizing volumes from 0 to 12 at increments of 1 (see Supplementary Table S1).

The optimum cutoff was then evaluated for REP predictive ability using binary logistic regression for multivariate analysis with various other predictors such as age, tumor location, EOR, ECOG performance status, sex, and SRI. These covariates were selected a priori. Additionally, IDH status was not included due to limited availability of this information and the expected predominance of IDH-wildtype tumors in this cohort. Patients that were missing data for MGMT methylation status were categorized as “unknown” for this category. A similar array of tests was used to identify the most significant, actionable, SRI cutoff by comparing various thresholds in increments of 1 week from surgery. Likewise, the most significant cutoff was evaluated on multivariate analysis. Hazard ratios and multivariate analysis of OS and PFS were obtained using Cox proportional hazards. Statistical significance was defined as a *P*-value <.05 and Bonferroni correction was applied.

## Results

### Clinical Outcomes

The median OS for all patients in this study was 18.1 months (15.802-20.398), with the median age being 61.5 years. The median PFS was 10.7 (9.199-12.335) months. The incidence rate for REP_2cc_ in this study was 166/318 (52.2%) and was relatively evenly distributed across various patient demographics (see Table 1). On Kaplan-Meier analysis using a Mantel-Cox test, presence of REP ≥ 2cc was statistically significantly associated with poorer survival outcomes (22.5 months vs 14.5 months). Increased age, worse ECOG performance status, MGMT methylation, and EOR were also significantly associated with poorer OS (*P* < .001). On multivariate analyses using a cox regression, presence of REP_2cc_ (hazard ratio [HR] [CI] = 1.459 [1.104-1.927]), increasing age (HR = 1.034 [1.020-1.049]), MGMT methylation status (unmethylated vs methylated; HR = 2.442 [1.792-3.327]), increasing ECOG (HR = 1.717 [1.463-2.015]) and smaller EOR (GTR vs STR: HR = 1.614 [1.165-2.236]) were all associated with poorer OS with statistical significance (see Table 2). Whereas REP (HR = 1.607 [1.210-2.133]) and MGMT methylation status (unmethylated vs methylated: HR = 2.290 [1.682-3.117]) were statistically significant predictors of poor PFS on both univariate and multivariate analysis (see Table 3).

**Table 1.** Patient characteristics

	REP_2cc_					
	No			Yes		
	*n*		%	*n*	%	*P*-value
Age		60.75 (median)	15.69 (IQR)	62.21 (median)	13.95 (IQR)	.172
Sex	Male	94	61.80%	98	59.00%	.609
	Female	58	38.20%	68	41.00%	
ECOG	0	74	48.7%	62	37.30%	.021
	1	61	40.1%	75	45.20%	
	2	12	7.9%	19	11.40%	
	3	5	3.3%	7	4.20%	
	4	0	0.00%	3	1.80%	
Tumor location	Frontal	53	34.9%	50	30.10%	.825
	Occipital	10	6.6%	12	7.20%	
	Parietal	28	18.4%	36	21.70%	
	Temporal	59	38.8%	64	368.60%	
	Multifocal	2	1.3%	4	2.40%	
MGMT methylation	Methylated	49	32.2%	63	38.00%	.296
	Unmethylated	84	55.3%	90	54.20%	
	Unknown	19	12.5%	13	7.80%	
Extent of resection	GTR	85	55.9%	57	34.3%	<.001
	NTR	30	19.7%	43	25.9%	
	STR	37	24.3%	66	39.8%	
Tumor volume	Pre-op	23.21 (median)	36.92 (IQR)	25.18 (median)	31.84 (IQR)	.700
	Post-op	1.76	2.98	4.03	8.76	.048
	Pre-RT	2.54	2.46	13.75	16.11	<.001
Days from resection to planning MRI		23 (median)	8 (IQR)	24 (median)	11 (IQR)	.005
Days from resection to first day of RT		32 (median)	9 (IQR)	33 (median)	13 (IQR)	.018

**Table 2.** Predictors of overall survival following resection

	Univariate	Multivariate	
	*P*-value		Hazard ratio
REP_2cc_	**<.001**	**0.008**	**1.459**
Age	<**.001**	**<0.001**	**1.034**
Sex	.258	0.083	
ECOG	**<.001**	**<0.001**	**1.717**
Tumor location	.093	0.097	
Frontal			Ref
Occipital		0.198	
Parietal		0.179	
Temporal		0.965	
Multifocal		0.052	
MGMT methylation	**<.001**	**<0.001**	
Methylated			Ref
Unmethylated		**<0.001**	**2.442**
Unknown		0.922	
Extent of resection	**<.001**	**0.012**	
GTR			Ref
NTR		0.053	
STR		**0.004**	**1.614**
Days from resection to pre-RT MRI	.141	0.320	

Note: Bolded values indicate statistically significant associations.

**Table 3.** Predictors of progression free survival following resection

	Univariate	Multivariate	
	*P*-value		Hazard ratio
REP_2cc_	**.002**	**<0.001**	**1.607**
Age	.343	0.195	
Sex	.775	0.949	
ECOG	.507	0.397	
Tumor location	.059	0.062	
Frontal			Ref
Occipital		0.763	
Parietal		0.393	
Temporal		**0.044**	**1.375**
Multifocal		0.208	
MGMT methylation	**<.001**	**<0.001**	
Methylated			Ref
Unmethylated		**<0.001**	**2.290**
Unknown		0.961	
Extent of resection	**.036**	0.281	
GTR			Ref
NTR		0.134	
STR		0.272	
Days from resection to pre-RT MRI	.766	0.128	

Note: Bolded values indicate statistically significant associations.

### Defining the Optimum Threshold of REP

All cutoffs of change in tumor volume between post-operative MRI and planning MRI except for 15% and 20% maintained statistical significance in univariate and multivariate testing with OS (*p*_Bonferroni_ < 0.00313). Overall, volume thresholds were superior to the percentage change in tumor volume between post-op and planning MRI. A residual of 2 cc was the lowest cutoff that remained statistically ­significant after Bonferroni correction (*P* = .002) (see Supplementary Table S2). Given that the trends of HRs and *P*-value were consistent across every threshold, an increase in tumor volume by at least 2 cc from post-operative to pre-RT was selected as it was the most significant and clinically appreciable threshold for REP. Separate analysis of the impact of satellite progression (*n* = 25) on REP showed that even when excluding these patients and only including those that locally recurred (*n* = 141), the definition of REP remained statistically significantly associated with OS on multivariate analysis.

### Predictors for REP

Using a chi-square or binary logistic regression analysis, patient characteristics such as EOR (GTR vs STR; *P* = .022; OR [CI] = 1.711 [1.079-2.713]), days from surgery to planning MRI (*P* = .005; OR = 1.040 [1.012-1.069]) and ECOG (*P* = .021; OR = 1.381 [1.051-1.816]) were identified as predictors of REP_2cc_. Other characteristics such as sex, age, MGMT methylation status, and tumor location did not reach statistical significance (see Table 1). A postoperative residual of 5 cc was identified as the most significant (*p*_Bonferroni_ < 0.00385), actionable cutoff associated with REP_2cc_ (*P* = <.001; OR = 6.038 [2.452-14.865]) and had a distribution of 279 patients with < 5 cc and 39 patients with ≥ 5 cc of residual tumor volume on postoperative MRI (see Supplementary Table S1).

The median time from resection to RT planning MRI was 23 (22-25) days for those without REP_2cc_ and 24 (23-26) days for those with REP_2cc_ making roughly 3 weeks the average interval. On chi-square tests, the most significant, defined as *p*_Bonferroni_ < 0.00833, interval was 6 weeks (*P* = .001; OR = 13.91 [1.806-107.090]) (see Supplementary Table S3). A separate subgroup analysis based on SRI cutoff of 3 weeks showed that in patients with intervals lower than 3 weeks, REP had a mOS of 13.3 months compared with 20.5 months for those without REP (*P* = .017). In patients with intervals > 3 weeks, those with REP had a mOS of 14.9 compared to 25.5 for those without REP. Additionally, the median time for resection to initiation of RT was 32 (30-33) days for those without REP_2cc_ and 33 (31-35) days for those with REP_2cc_.

On a binary logistic regression for predictive ability of various patient characteristics, both residual tumor of 5 cc (*P* = .004; OR = 4.785 [1.665-13.754]) as well as SRI_6weeks_ (*P* = .007; OR = 18.200 [2.223-149.00]) remained statistically significant (see Table 4). EOR also remained statistically significant for GTR vs NTR (*P* = .007; OR = 2.284 [1.254-4.159]). Characteristics such as tumor location, ECOG performance, MGMT methylation status, sex, and age were all not found to be statistically significant predictors.

**Table 4.** Predictors of REP_2cc_

	*P*-value	OR	CI
Residual tumor (5 cc)	**.004**	4.785	1.665-13.754
Days to plan MRI (6w)	**.007**	18.200	2.223-149.00
Tumor location	.711	-	-
EOR	**.018**	-	-
GTR	ref	-	-
NTR	**.007**	2.284	1.254-4.159
STR	.081	-	-
ECOG	.255	-	-
Sex	.417	-	-
MGMT methylation	.324	-	-
Age	.716	-	-

Note: Bolded values indicate statistically significant associations.

## Discussion

With REP defined as either a new enhancement following GTR or an increase in T1-enhancing volume from post-operative of at least 2 cc, REP_2cc_ had an incidence rate of 52.2%. This incidence is higher than reported in some prior studies, including Wee et al.^13^ This may be attributable to differences in methodology, as the current study uses a lower, volumetric threshold for defining REP and did not assess functional imaging, which may increase sensitivity to early progression. Prior studies like Wee et al had varying definitions for REP, including requirements for new or progressing enhancement as well as assessment of functional imaging.

The study showed that certain characteristics such as residual tumor volume >5 cc on post-operative MRI and the length of surgery-to-RT interval > 6 weeks were significant predictors of REP_2cc_. While the median SRI in this cohort was approximately 3-4 weeks, the 6-week threshold accounts for a smaller subset of patients with delayed treatment in which there was an increased risk of REP.

However, interval time did not completely explain presence of REP as seen in the multivariate analysis and subgroup analysis. Due to the limited number of patients without REP in the SRI >6 weeks group (1/12), subgroup analyses using this threshold were underpowered. Hence, we also assessed an SRI cutoff of 3 weeks as this was closer to the median and allowed for a more balanced subgroup comparison to assess effect of SRI on REP. This analysis shows that regardless of SRI, REP was still associated with poorer outcomes. An additional subgroup analysis based on EOR was also performed, and although REP was associated with poorer median OS in every group, the differences were not statistically significant.

The prognostic importance of extent of resection in glioblastoma has been well established, with prior studies demonstrating improved survival following more complete resection of contrast-enhancing tumor.^16^^,^^17^ A prior study by Chaichana et al analyzing the relationship between residual tumor volume and OS had also found 5 cc to be the maximal residual volume significantly associated with survival after contouring volumes of tumor on post-operative scans.^15^ The results of this study suggest that not only is residual tumor volume associated with worse survival but also associated with incidence of REP_2cc_. This indicates that the prognostic ability of residual tumor volume may, in part, be mediated by its association with REP. Additionally, this reinforces that surgical treatment aiming for < 5 cc of residual tumor can significantly impact patient outcomes, as this can be associated with lower likelihood for early progression.

Although some prior studies have failed to identify a significant association between SRI and REP, this study found SRI to be significantly associated with both REP_2cc_ and OS as a continuous variable of days from resection to planning MRI. To identify a potential actionable recommendation, this interval was dichotomized in increments of weeks from surgery, beginning with 2 weeks from surgery to 7 weeks (no patient in this study that met inclusion criteria had an interval shorter than 1 week). While there was a consistent positive association between REP and SRI on cutoffs ranging from 2-6 weeks, this was not observed at 7 weeks which instead showed a negative association (see Supplementary Figure S1b). This may in part reflect the small number of patients at longer SRI thresholds, resulting in less stable estimates. For example, only 7 patients had an SRI >7 weeks compared with 15 who had an SRI >6 weeks, limiting the reliability of estimates at this cutoff. However, imbalanced sampling at longer thresholds does not fully explain these findings, as some intermediate thresholds (3 weeks) did not reach statistical significance despite balanced group sizes.

It is also possible that treatment timing was influenced by postoperative clinical or radiographical urgency. Patients with a more aggressive disease were prioritized for earlier RT, whereas patients with a less concerning course experienced greater delays. As a result, this may attenuate the relationship between SRI and REP at longer, delayed thresholds. However, this does not fully account for the consistently positive ORs observed between 2 and 6 weeks as well as the relative increase in effect size at the 6-week threshold.

Ultimately, the 6-week threshold appeared to provide a more clinically informative separation in this cohort given the overall positive association in ORs seen in thresholds from 2 to 5 weeks as well as the higher OR observed at the 6-week threshold.

Despite what this association may imply, some prior studies have shown there to be no clinical benefit to earlier initiation of RT+TMZ. Since current guidelines and trends show that the interval between resection and planning MRI ranges from 4 to 6 weeks, the results of this study instead serve to reinforce the importance of timing intervention and that although very early RT may not negatively influence outcomes, delays of RT beyond 6 weeks may increase the likelihood of developing detectable REP.

None of the other potential predictors of REP_2cc_ such as EOR, ECOG, sex, age, and MGMT methylation were significant, and these factors were independently prognostic for OS on multivariate analysis. Also of note, there were instances in this study of GTR with later REP_2cc_ on RT-planning MRI. This suggests that although residual tumor volume could be associated with increased risk for REP, it isn’t a necessity, as suggested by the weaker significance and OR when testing any amount of residual with REP_2cc_ on chi-squared analysis (see Supplementary Table S1).

Based on the screening of various REP cutoffs, 2 cc was chosen, as there were consistent *P*-value and HRs throughout the various thresholds. The lowest threshold that remained statistically significant was selected to balance sensitivity for REP with clinical interpretability, corresponding to an identifiable volume (roughly a 1.5 cm diameter sphere). This definition may also help minimize the influence of nonmalignant enhancement etiologies, such as postoperative enhancement, variation in imaging protocols/techniques, and slice thickness variability, which could substantially vary between post-op scans and planning MRI. However, these factors were not directly evaluated.

REP_2cc_ as defined in this study was significantly associated with both poorer OS and decreased PFS, which adds to prior studies that only found an association with PFS. In addition, on cox regression analysis for OS, when controlling for various confounders such as ECOG performance status, MGMT methylation status, age, and EOR, REP remained a significant prognostic factor in addition to the aforementioned factors.

Limitations of this study included factors such as lack of patients who underwent only biopsy (principally because of a lack of postoperative MRI) and exclusion of non-enhancing tumors. In fact, there could be a relationship between non-enhancing tumors and REP, although not evaluated in this study. Among non-enhancing gliomas, given the difficulty in delineating non-enhancing tumors from tumor-related edema in the perioperative period and the lack of standardized methods, we did not attempt this approach, although it could be considered in future work or with advanced imaging. To avoid interobserver variability and minimize subjectivity of analysis, a single trained investigator blinded to patient outcomes contoured all the cases in this study, and conflicts, such as indeterminate enhancement, were clarified with a second reviewer. However, we were not able to assess intra-observer variability, which in addition to inter-observer variability assessment, could further strengthen the significance of the 2 cc threshold. Future studies could also benefit from incorporating automated or semi-automated segmentation tools to improve consistency across multiple centers and increase viability as a scalable prognostic tool.

Additionally, given that the study period ranged from 2016 to 2023, a number of cases are largely defined by the 2016 WHO criteria, which looked only at histological markers as opposed to using the 2021 WHO classification, which includes additional clinical and molecular considerations. This may limit generalizability to current clinical practice. Also of note, given the relatively small volumes of cutoffs and thresholds tested and selected, estimations of very small volumes can significantly impact these findings, particularly when considering changes as a percentage. This is especially apparent when slice thicknesses vary to such an extent that some may span several millimeters, leading to significant estimations in actual tumor volume. With a threshold at the minimal clinically appreciable level, a “background noise” of enhancement could prove challenging to delineate and we hoped to limit this with the strict and standardized definition of enhancement (ie limiting it to clear nodular enhancement, excluding blood products and postoperative changes). Another limitation was the inconsistent availability of functional MRI techniques, such as DWI and SWI, which some studies have posed may be more accurate in defining REP. Finally, as this study is a retrospective study, there is bias in relationships tested, and conclusions drawn may not indicate a true and direct causal relationship between variables like REP and OS. Despite these limitations, this study has controlled confounders through multivariate analyses and the use of a single reviewer, tested various cutoffs to determine the most precise outcome, controlled for as much variance as possible with strict inclusion criteria and a consistent standardized approach to segmentations for all cases, and provides data for potential studies to build upon in a path toward better-informed clinical practices.

## Conclusion

Glioblastoma is highly malignant and relatively common among brain tumors. REP of glioblastoma is a significant prognostic factor herein defined by a 2-cc increase in tumor enhancement immediately prior to RT and/or a new satellite lesion. Postoperative residual tumor volume (over 5 cc) and delays in postoperative RT beyond 6 weeks were independently associated with REP, which in turn is associated with reduced OS. This study identifies not only potential surgical goals to minimize REP occurrence but also establishes a clear prognostic factor for future patient risk stratification.

## Supplementary Material

vdag153_Supplementary_Data

## Data Availability

The data that support the findings of this study are not publicly available due to privacy or ethical restrictions but are available from the corresponding author upon reasonable request and institutional approval.
