# Supplementary material for: Defining rapid early progression in glioblastoma—A volumetric approach
Source: Neurooncol Adv. 2026 Jun 8;8(1):vdag153. doi: 10.1093/noajnl/vdag153 (PMC13318312; doi:10.1093/noajnl/vdag153)
Supplement: vdag153_Supplementary_Data [file vdag153_supplementary_data.docx]

**Supplementary Material for “Defining Rapid Early Progression in Glioblastoma – A Volumetric Approach”**

Vikram Munlapudi, BS^1^, Jacob Guzzino, BS^1^, David J. Crompton, MD^1^, Shubhi Agarwal, MD ^1^, Eric J. Lehrer, MD^2^, Shelby Kern, PA-C, MS^2^, Aaron Bogan, MA^3^, Sujay A. Vora, MD^4^, Alfredo Quinones-Hinojosa, MD^5^, Terry C. Burns, MD, PhD^6^, Wendy J. Sherman, MD^7^, Paul D. Brown, MD^2^, Nadia Laack, MD^2^, Jennifer Peterson MD^1^, Joon H. Uhm, MD^8^, Michael W. Ruff, MD^8^, Ugur T. Sener, MD^8^, William G. Breen, MD^2^, Daniel M. Trifiletti, MD^1^

^1^ Mayo Clinic Florida, Department of Radiation Oncology, Jacksonville, FL, United States

^2^ Mayo Clinic Rochester, Department of Radiation Oncology, Rochester, MN, United States

^3^ Mayo Clinic Arizona, Department of Research Biostatistics, Phoenix, AZ, United States

^4^ Mayo Clinic Arizona, Department of Radiation Oncology, Phoenix, AZ, United States

^5^ Mayo Clinic Florida, Department of Neurosurgery, Jacksonville, FL, United States

^6^ Mayo Clinic Rochester, Department of Neurosurgery, Rochester, MN, United States

^7^ Mayo Clinic Florida, Department of Neurology and Oncology, Jacksonville, FL, United States

^8^ Mayo Clinic Rochester, Department of Neurology and Oncology, Rochester, MN, United States

**Corresponding Author:**

Daniel M. Trifiletti, MD

Department of Radiation Oncology

Mayo Clinic

4500 San Pablo Road South, Jacksonville, FL, 32224

Email: trifiletti.daniel@mayo.edu

ORCID: 0000-0002-9150-2916

**Supplementary Methods:**

*Statistical Analysis*

Various thresholds of changes in post-operative tumor volume between 1-10cc and at 5% intervals from 5-20% (26% was included as the median percent change in tumor volume) were tested using both univariate and multivariate analysis using Cox-proportional hazards test via IBM SPSS Statistics. The chosen cutoff was then used to define REP in further statistical tests to identify actionable predictors using chi-square and binary logistic regression analysis.

**Supplementary Results:**

*Predictors for REP*

Supplementary Table 1 lists the p-values and OR of REP, as defined as a change in tumor volume of >2cc in the surgery-to-radiotherapy interval (SRI), in patients with various cutoffs of post-operative residual volume. Supplementary Table 3, similarly, lists p-values and OR of REP for various surgery to radiotherapy intervals.

| **Supplementary Table 1:** Association between postoperative residual volume and REP. | | | | | |
| --- | --- | --- | --- | --- | --- |
|  |  |  |  | **Count** | |
|  | *p-value* | *OR* | *CI* | *Under* | *Above* |
| Any | 0.022 | 1.711 | 1.079-2.713 | 199 | 119 |
| 1cc | 0.002 | 2.179 | 1.319-3.598 | 225 | 93 |
| 2cc | <0.001 | 2.689 | 1.524-4.743 | 247 | 71 |
| 3cc | <0.001 | 3.738 | 1.930-7.276 | 262 | 56 |
| 4cc | <0.001 | 3.55 | 1.735-7.264 | 271 | 47 |
| **5cc** | **<0.001** | **6.038** | **2.452-14.865** | **279** | **39** |
| 6cc | <0.001 | 5.588 | 2.261-13.812 | 281 | 37 |
| 7cc | 0.001 | 4.113 | 1.633-10.361 | 288 | 30 |
| 8cc | 0.001 | 4.492 | 1.656-12.183 | 291 | 27 |
| 9cc | 0.0037 | 4.027 | 1.472-11.018 | 293 | 25 |
| 10cc | 0.006 | 3.8 | 1.382-10.447 | 294 | 24 |
| 11cc | 0.01 | 3.947 | 1.289-12.083 | 298 | 20 |
| 12cc | 0.093 | 2.626 | 0.818-8.429 | 303 | 15 |

*Defining the optimum threshold of REP*

Supplementary Table 2 provides the p-values and hazard ratios for each of the cutoffs including the 2cc change that was selected in the study.

| **Supplementary Table 2: Overall Survival based on varying REP cutoffs** | | | | | | | | | | | |  |
| --- | --- | --- | --- | --- | --- | --- | --- | --- | --- | --- | --- | --- |
|  | **Univariate** | | **Multivariate** | | | | **CI** | | **Count** | | | |
|  | *p-value* | | | | *Hazard Ratio* | | *Lower* | *Upper* | *Under* | | *Above* | |
| Any | <0.001 | | 0.017 | | 1.495 | | 1.075 | 2.076 | 112 | | 206 | |
| 1cc | <0.001 | | 0.004 | | 1.513 | | 1.141 | 2.006 | 139 | | 179 | |
| **2cc** | <0.001 | | 0.002 | | 1.545 | | 1.177 | 2.028 | 152 | | 166 | |
| 3cc | 0.004 | | 0.004 | | 1.479 | | 1.13 | 1.938 | 159 | | 159 | |
| 4cc | 0.002 | | <0.001 | | 1.568 | | 1 | 2.049 | 165 | | 153 | |
| 5cc | 0.0026 | | 0.001 | | 1.556 | | 1.19 | 2.033 | 169 | | 149 | |
| **6cc** | <0.001 | | <0.001 | | 1.775 | | 1.352 | 2.331 | 175 | | 143 | |
| **7cc** | <0.001 | | <0.001 | | 1.741 | | 1.326 | 2.285 | 178 | | 140 | |
| **8cc** | <0.001 | | <0.001 | | 1.708 | | 1.302 | 2.242 | 179 | | 139 | |
| 9cc | 0.002 | | <0.001 | | 1.69 | | 1.284 | 2.224 | 185 | | 133 | |
| 10cc | 0.001 | | <0.001 | | 1.745 | | 1.322 | 2.302 | 186 | | 132 | |
| 5% | 0.001 | | 0.009 | | 1.391 | | 1.01 | 1.916 | 115 | | 203 | |
| 10% | 0.001 | | 0.056 | | - | | - | - | 118 | | 200 | |
| 15% | 0.001 | | 0.044 | | 1.376 | | 1.008 | 1.88 | 120 | | 198 | |
| 20% | 0.002 | | 0.053 | | - | | - | - | 122 | | 196 | |
| 26.0% | 0.002 | | 0.053 | | - | | - | - | 125 | | 193 | |
| **Supplementary Table 3:** Association between SRI and REP. | | | | | | | | | | | |  |
|  | |  | |  | |  | | **Counts** | | | |  |
|  | | *p-value* | | *OR* | | *CI* | | *Under* | | *Above* | |  |
| 2 weeks | | 0.092 | | 3.019 | | 0.786-11.594 | | 11 | | 307 | |  |
| 3 weeks | | 0.419 | | 1.21 | | 0.762-1.922 | | 110 | | 208 | |  |
| 4 weeks | | 0.039 | | 1.669 | | 1.024-2.722 | | 223 | | 95 | |  |
| 5 weeks | | 0.011 | | 2.637 | | 1.226-5.672 | | 282 | | 36 | |  |
| 6 weeks | | **0.001** | | 13.91 | | 1.806-107.090 | | 303 | | 15 | |  |
| 7 weeks | | **0.004** | | 0.508 | | 0.455-0.567 | | 309 | | 9 | |  |


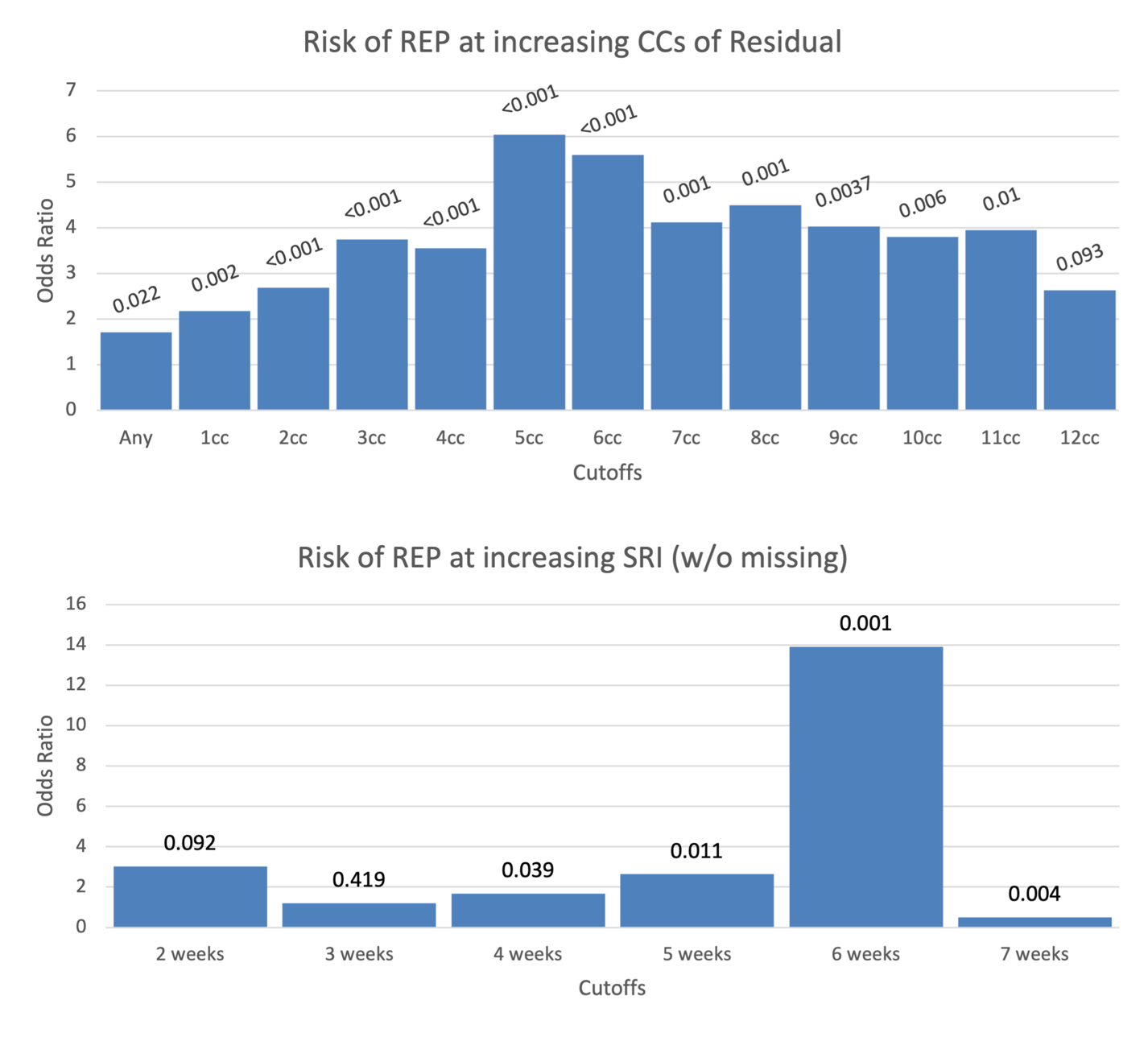


**Supplemental Figure 1:** Odds ratios for risk of REP at various cutoffs of A) residual and B) surgery-to-radiotherapy interval are graphed above. Data labels above each bar depict p-values for each respective cutoff.
